# The multifaceted functions of long non-coding RNA *HOTAIR* in neuropathologies and its potential as a prognostic marker and therapeutic biotarget

**DOI:** 10.1017/erm.2024.11

**Published:** 2024-04-29

**Authors:** Faraz Ahmad, Ravi Sudesh, Atheeq Toufeeq Ahmed, Mohanapriya Arumugam, Darin Mansor Mathkor, Shafiul Haque

**Affiliations:** 1 Department of Biotechnology, School of Bio Sciences (SBST), Vellore Institute of Technology (VIT), Vellore 632014, India; 2 Department of Biomedical Sciences, School of Bio Sciences (SBST), Vellore Institute of Technology (VIT), Vellore 632014, India; 3 Research and Scientific Studies Unit, College of Nursing and Allied Health Sciences, Jazan University, Jazan 45142, Saudi Arabia; 4 Gilbert and Rose-Marie Chagoury School of Medicine, Lebanese American University, Beirut, Lebanon

**Keywords:** brain injury, ceRNA, glioma, neurodegeneration, neuropsychological conditions

## Abstract

Long non-coding RNAs (lncRNAs) are progressively being perceived as prominent molecular agents controlling multiple aspects of neuronal (patho)physiology. Amongst these is the HOX transcript antisense intergenic RNA, often abbreviated as *HOTAIR*. *HOTAIR* epigenetically regulates its target genes via its interaction with two different chromatin-modifying agents; histone methyltransferase polycomb-repressive complex 2 and histone demethylase lysine-specific demethylase 1. Parenthetically, *HOTAIR* elicits trans-acting sponging function against multiple micro-RNA species. Oncological research studies have confirmed the pathogenic functions of *HOTAIR* in multiple cancer types, such as gliomas and proposed it as a pro-oncological lncRNA. In fact, its expression has been suggested to be a predictor of the severity/grade of gliomas, and as a prognostic biomarker. Moreover, a propound influence of *HOTAIR* in other aspects of brain heath and disease states is just beginning to be unravelled. The objective of this review is to recapitulate all the relevant data pertaining to the regulatory roles of *HOTAIR* in neuronal (patho)physiology. To this end, we discuss the pathogenic mechanisms of *HOTAIR* in multiple neuronal diseases, such as neurodegeneration, traumatic brain injury and neuropsychiatric disorders. Finally, we also summarize the results from the studies incriminating *HOTAIR* in the pathogeneses of gliomas and other brain cancers. Implications of *HOTAIR* serving as a suitable therapeutic target in neuropathologies are also discussed.

## Introduction

Long non-coding RNAs (lncRNAs) are RNAs which do not translate into peptides and have a length in excess of 200 nucleotides. These are widely present in multiple regions across the genome and are generally produced by RNA polymerase II-mediated transcription. Numerous species of lncRNA transcripts have been identified which differ greatly in their biogenesis as well as (patho)physiological functions. lncRNAs are a versatile group of RNAs which elicit widespread, but tissue- and cell type-specific distribution. Indeed, in the latest version of Gencode 43 (2022), more than 16 000 genes producing in excess of 100 000 transcripts are catalogued as lncRNAs (Ref. 1). The (patho)physiological functions of lncRNAs rely on their interaction with other biomolecular species, such as DNA, mRNA, proteins and other non-coding RNAs such as micro-RNAs (miRNAs). Accordingly, lncRNAs are known to play prominent roles in several biological phenomena.

lncRNAs are abundantly found in the central nervous system (CNS); both in neurons and glia. Recent research studies have recognized noticeable roles of various novel lncRNAs in gene expression regulation in CNS cells. As such, lncRNAs intricately and dynamically modulate a plethora of signalling pathways central to the (patho)physiological brain states. At the nuclear level, lncRNAs are known to modulate processes such as remodelling and organization of the chromatin structure, transcription initiation and progression, as well as splicing and export of mRNAs. When present in the cytoplasm, lncRNAs regulate stability, localization and translation of mRNAs; function as molecular sponges for other RNAs and proteins and may even participate in post-translational protein modifications (Ref. 2). Readers are directed to recent reviews that have investigated the interesting roles of different lncRNAs in CNS pathologies, including tumourigenesis (Refs 3, 4, 5, 6).

**Figure 1.**
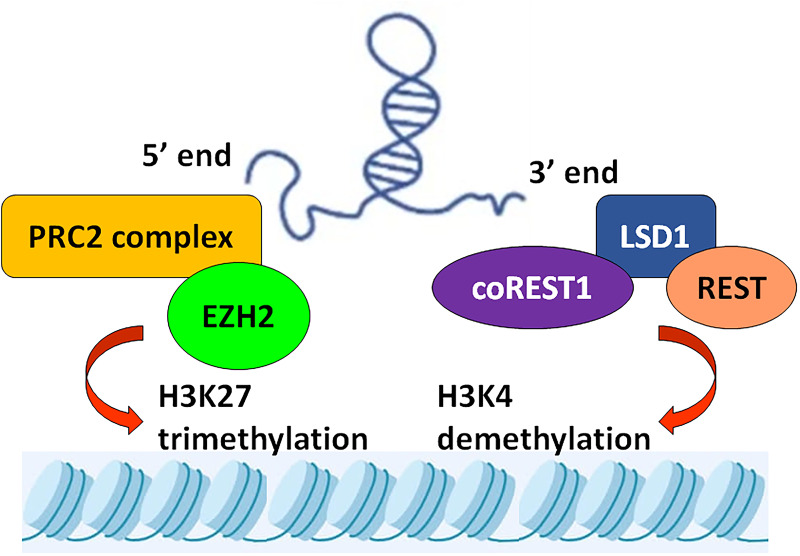
Mechanism underlying *HOTAIR*-mediated epigenetic regulation of target genes. While its 5′ end is thought to bind and regulate the H3K27 trimethylation activity of the PRC2/EZH2, the 3′ end of *HOTAIR* is involved in the modulations of the H3K4 demethylation activity of LSD1–REST–CoREST.

HOX transcript antisense intergenic RNA (*HOTAIR*) is an lncRNA species transcribed from the antisense strand of the *HOXC* gene which is localized on chromosome 12q13.13 featuring clustered HOX genes (*HOXA*, *B*, *C* and *D*). *HOTAIR* has multiple isoforms ranging from 4 to 8 exons which are spliced and polyadenylated. Discovered in 2007, *HOTAIR* was proposed as a novel lncRNA with an interesting ability to regulate the expression of target genes by trans-silencing mechanisms. *HOTAIR* interacts with histone methyltransferases, polycomb-repressive complex 2 (PRC2) through the latter's catalytic subunit, enhancer of Zeste homologue 2 (EZH2), and can modulate its trimethylation activity at lysine 27 of histone H3 (H3K27me3). This allows *HOTAIR* to alter epigenetic transcriptional regulators for many target genes (Ref. 7). Moreover, *HOTAIR* may also act as a scaffold by forming a complex with lysine-specific demethylase 1 (LSD1), repressor element 1 silencing transcription factor (REST), REST corepressor 1 (CoREST1) and modulate the latter's function of demethylation of histone H3 lysine-4 dimethylation (H3K4me2) (Ref. 8). Interestingly, 5′ domain of *HOTAIR* is thought to bind to and modulate PRC2/EZH2 activity, whereas its 3′ domain may be involved in the modulation of LSD1–REST–CoREST signalling. Both these mechanisms are understood to contribute to the epigenetic alteration of the expressions of target genes by *HOTAIR* (Fig. 1).

Multiple lines of evidence suggest that *HOTAIR* exerts a variety of effects during the pathogeneses of malignancies and inflammatory diseases (reviewed in Refs 9, 10). Increased *HOTAIR* expression has been evidenced to be associated with genome-wide retargeting of PRC2 and LSD1–CoREST1, leading to altered epigenetic regulation of gene expression. These changes have been evidenced to severely aggravate tumour cell invasiveness and metastases. On the contrary, *HOTAIR* repression has been shown to attenuate tumourigenesis and cancer progression, indicating that it might serve as a prominent mechanism in cancer pathogenesis (Ref. 11). It is hardly surprising then that increased *HOTAIR* levels are strongly linked with higher grades of the tumours and their severity, and poor survival in cancers (Ref. 12). Indeed, *HOTAIR* may represent amongst the most expansively studied oncologically relevant lncRNAs which is repeatedly observed to be dysregulated in various cancer types.

This comprehensive review aims to accumulate and summarize the evidences of the involvement of *HOTAIR* in multiple pathogenic mechanisms central to the progression of brains cancers and other CNS disorders. The authors initiate the discussion with the general pathways through which *HOTAIR* may exert its (patho)physiological effects in the neuronal perspective, and then proceed to review the implications of *HOTAIR* in brain (patho)physiology, including in neurodegeneration (e.g. Alzheimer's disease (AD), Parkinson's disease (PD) and multiple sclerosis (MS)), physical brain injuries, diabetic neuropathy, neuropsychological conditions and brain cancers. Finally, potential utilities of *HOTAIR* as a tremendous prognostic agent and therapeutic biotarget are outlined in detail.

## Overview of neuronal pathways affected by *HOTAIR*

**Figure 2.**
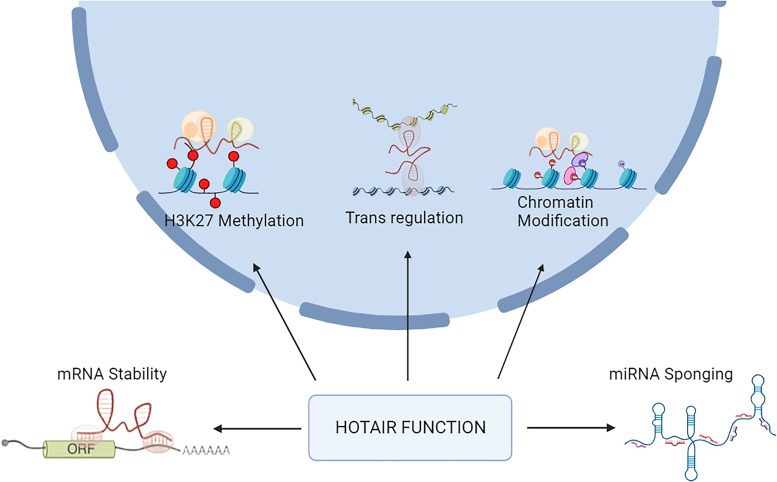
Multifaceted actions of *HOTAIR* in neuronal (patho)physiology. In addition to chromatin remodelling and epigenetic alterations, *HOTAIR* also impacts mRNA stability and can act as a molecular sponge against specific miRNAs.

Although *HOTAIR* lncRNA is widely expressed throughout the body, brain is considered to elicit high expression levels (Ref. 13). Interestingly, the expression of *HOTAIR* shows appreciable brain region specificity and dynamicity, indicating that it likely regulates several features of neuronal development and (patho)physiology. Figure 2 summarizes the mechanisms by which *HOTAIR* can alter neuronal functions. *HOTAIR* was the first lncRNA which was shown to modulate the expression of target genes in a trans-silencing manner. As discussed, gene regulation mediated by *HOTAIR* is through its interaction with epigenetic regulators, histone methyltransferase PRC2 and histone demethylase LSD1. *HOTAIR* can epigenetically repress a plethora of target genes and hence contribute to the underlying pathogenic mechanisms such as inflammatory responses, cell apoptosis, oxidative stress and damage and autophagy in the CNS. With regards to tumourigenesis, *HOTAIR* influences cell migration/invasion, metastasis, drug resistance and epithelial-to-mesenchymal transition (EMT) cascades.

### Neuronal development

Phosphorylation of proteins is a widespread cellular mechanism controlling precise spatio-temporal aspects of protein functions/interactions. Kaneko *et al*. indicated that *HOTAIR*–EZH2 interaction may be controlled by alterations in phosphorylation status of EZH2 in a site-specific manner. Thus, *HOTAIR* was reported to bind to EZH2 with greater affinity when the latter was phosphorylated at T-345 (Ref. 14). In view of this study, and given the proposed regulatory actions of EZH2 in neuronal differentiation and recovery following ischaemic stroke (Ref. 15), it is stimulating to theorize probable roles of *HOTAIR* in neuronal differentiation. Indeed, in silico and biochemical data indicate that inhibition of the *HOTAIR*–EZH2 interaction mediated by amplified miRNA-141 expression is linked to expressional changes in brain-derived neurotrophic factor (BDNF), and the differentiation of amniotic epithelial stem cells into dopaminergic neuron-like cells (Ref. 16). Similarly, Khani-Habibabadi *et al*. have reported that *HOTAIR* is a regulator of oligodendrocyte precursor cell differentiation (Ref. 17).

### Neuroinflammatory signalling

Neuroinflammatory pathways are driven by enhanced production of proinflammatory signalling molecules and secondary messengers (cytokines, chemokines, etc.) by immune cells such as endothelial cells, astrocytes and microglia. Often, these responses are associated with redox dyshomoeostasis and oxidative damage. Pro-inflammatory and pro-oxidant signalling is aberrantly activated in almost all neurological disorders. Recent data show that lncRNAs such as *HOTAIR* contribute immensely to the modulation of both pro-oxidant and pro-inflammatory pathways in CNS (patho)physiology. Indeed, activated microglia which elicit upregulated expression of *HOTAIR* have been evidenced to excessively produce inflammatory factors (Ref. 18). Overexpression of *HOTAIR*, and the consequent high mobility group box 1- and nuclear factor kappa B (NF-*κ*B)-dependent activation of pro-inflammatory, oxidative and apoptotic pathways may contribute to the pathogenic events in spinal cord ischaemia–reperfusion injury (Ref. 19).

### Cell signalling

*HOTAIR* exerts changes in protein functions and cellular signalling cascades through post-translational modifications, such as ubiquitin-mediated protein degradation. For instance, *HOTAIR* may bind to the RNA-binding domains of E3 ubiquitin ligases, DAZ-interacting zinc finger 3 and Mex-3 RNA-binding family member B (Mex3b). This interaction has been shown to result in facilitating their ubiquitination actions, resulting in proteolysis of target proteins, such as ataxin-1 and snurportin-1 which are involved in cellular senescence pathways (Ref. 20). It should be noted here that ataxin-1 is a crucial regulator of developmental pathways in the brain and its post-translational modifications and mutations in its gene are thought to be associated with CNS dysfunctions such as spinocerebellar ataxia type 1 (Ref. 21). Similarly, interactions of *HOTAIR* with Mex3b and the consequent alterations in functions of suppressor of mothers against decapentaplegic family member 4 and nucleoside diphosphate linked moiety X-type motif 3 (Ref. 22) may suggest potential participation of *HOTAIR* in multiple neurobiological processes.

## *HOTAIR* in brain (patho)physiology

**Figure 3.**
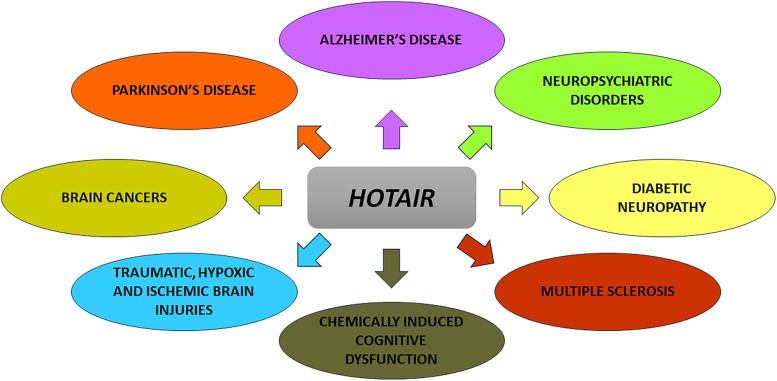
Involvement of *HOTAIR* in neuronal disorders. Owing to its multimodal regulation of neuronal (patho)physiology, *HOTAIR* is implicated in several brain pathologies, including AD, PD, MS, neuropsychiatric disorders, traumatic, hypoxic and ischaemic brain injuries, diabetic neuropathy/retinopathy, chemically induced cognitive decline and gliomas and other brain cancers.

Numerous studies have concentrated on the involvement of miRNAs in multiple brain pathologies. On the other hand, a similar comprehensive understanding of lncRNAs' functions in neuropathologies has largely been lacking. Nevertheless, during the last few years, attention has been focused on lncRNAs and their pathogenic roles in brain disorders, such as neurodegenerative diseases (Ref. 23). Below, we comprehensively discuss the evidences for the involvement of *HOTAIR* in varied kinds of CNS disorders. These include neurodegenerative states of AD, PD and MS, traumatic, ischaemic and hypoxic injuries, diabetic neuropathy, neuropsychological deficits and finally gliomas and other brain cancers (Fig. 3).

### Alzheimer's disease

In one of the earliest studies evaluating the expressional changes of lncRNAs in AD pathology, Lee *et al*. performed microarray analyses and reported appreciable dysregulations in *HOTAIR* levels in transgenic animal models; the 3xTg-AD mice (Ref. 24), which elicits overexpression of mutant forms of amyloid precursor protein (APP), presenilin-1 (PS-1) and microtubule-associated protein tau proteins. In another transgenic AD model, the APP/PS1 mice, implementation of voluntary exercise regimens was found to result in repression of *HOTAIR* expression. The diminishment of the sponging effects of *HOTAIR* on miR-130a-3p was proposed to be associated with the voluntary exercise-induced recovery of memory functions in the Morris water maze test and attenuation of pro-inflammatory responses in these mice (Ref. 25).

*HOTAIR* may also repress the levels of cyclin-dependent kinase 5 (Cdk5), possibly through its modulatory effects on the miR-15/107 pathway. Cdk5 is known as one of the central mediators of tau and amyloid pathologies and is severely hyper-activated in AD (Ref. 26), which may, at least in part, be dependent on the altered expression levels of lncRNAs, such as *NEAT1* and *HOTAIR*. In support of this idea, *HOTAIR* has been observed to elicit brain region-specific alterations in AD subjects, compared with controls (Ref. 27). Altered levels of *HOTAIR* have also been reported in serum samples of AD patients by Lu *et al*. In their study, increased serum expression of *HOTAIR* showed close associations with cognitive and memory impairments, as assessed by the Mini-Mental State Examination and Alzheimer's Disease Assessment Scale-Cognitive tests, leading the authors to propose serum *HOTAIR* expression as a probable diagnostic/prognostic biomarker for AD. Indeed, a 3-month long bicycle training regimen significantly reduced the serum levels of *HOTAIR* with concomitant attenuation of AD-linked cognitive impairment (Ref. 28). In spite of these data supporting a probable role of *HOTAIR* in AD pathogenesis, the exact identities of the molecular and cellular pathways have remained obscure and might represent an interesting area of research in the coming years.

### Parkinson's disease

A number of studies have implicated hyperactivation of *HOTAIR* in the pathogenesis of PD (Ref. 29). Multiple miRNAs have been proposed to be involved in the *HOTAIR*-harbouring competing endogenous RNA (ceRNA) network loops in PD (Ref. 30). The first studies proclaiming a role for *HOTAIR* in PD relied upon a chemically induced *N*-methyl-4-phenyl-1,2,3,6- tetrahydropyridine-mediated mouse model, and an in vitro PD model composed of *N*-methyl-4-phenylpyridinium (MPP^+^)-challenged SH-SY5Y cells. It was observed that in both these PD models, there was a significantly amplified expression of *HOTAIR* lncRNAs, in complementation with enhanced levels of leucine-rich repeat kinase 2 (LRRK2), a proteinaceous player strongly implicated in the disease pathogenesis (Ref. 31). Further, siRNA-induced knockdown of *HOTAIR* was found to be robustly neuroprotective, and resulted in attenuation of LRRK2 induction and reduced neuronal death (Refs 32, 33). In addition, altered sponging of miR-126-5p as a result of aberrant *HOTAIR* activity may also underlie promotion of PD phenotype, possibly through the mediation of RAB3A interacting protein (RAB3IP), a Rab-specific guanine nucleotide exchange factor which has prominent regulatory roles in synaptic physiology. Interestingly, siRNA-mediated repression of the *HOTAIR*–miR-126-5p–RAB3IP axis has been evidenced to result in inhibition of cellular apoptosis of tyrosine hydroxylase-positive cells, as well as in reduction in the number of *α*-synuclein-positive cells, concomitantly with appreciable diminution of PD-linked motor and cognitive decline (Ref. 34).

Altered activities of other target miRNAs have also been proposed as mediators of PD-promoting actions of *HOTAIR*. For instance, aberrant elevation in *HOTAIR* levels in dopaminergic neurons was observed to induce autophagy-associated genes (lysosomal-associated membrane protein type I and II, and microtubule associated protein-1 light chain 3 beta LC3B-I/LC3B-II ratio) in PD mice, probably via activation of the miR-221-3p–neuronal pentraxin II (NPTX2) pathway (Ref. 35). *HOTAIR* has also been reported to stimulate neuronal damage and Nod-like receptor (NLR) family pyrin domain containing 3-facilitated pyroptosis in PD via its sponging actions against miR-326, and the consequent stimulation of ELAV-like RNA binding protein 1 (ELAVL1) expression (Ref. 36). Further, Zhao *et al*. have postulated the involvement of the *HOTAIR*–miR-874-5p–autophagy-related protein 10 (ATG10) cascade in the induction of pro-inflammatory (elevated levels of interleukins: IL-6 and IL-1*β*, and tumour necrosis factor-alpha (TNF-*α*□), pro-apoptotic (upregulated levels of Bax protein and repressed expression of Bcl-2 protein) and pro-oxidative (enhanced cellular reactive oxygen species or ROS and diminished antioxidant activity) signalling in an MPP^+^-induced cellular model of PD (Ref. 37). More recently, it has been shown that *HOTAIR*-mediated sponging of miR-221-3p has direct effects on *α*-synuclein expression, resulting in the latter's high levels and consequent anomalous aggregation (Ref. 38).

### Multiple sclerosis

In support of the probable evidences of *HOTAIR* signalling in immunomodulation during demyelination and in the pathogenesis of MS (Ref. 39), the efficiencies of vitamin D3-reliant therapeutic strategies administered to MS patients has been reported to be because of their abilities, at least in part, to rescue the increased levels of *HOTAIR*. Thus, Pahlevan Kakhki *et al*. observed substantial upregulation of *HOTAIR* expression in peripheral blood mononuclear cells (PBMCs) of MS patients. Conversely, vitamin D3 supplementation could abolish the aberrant activation of *HOTAIR*. Further, vitamin D3's role in modulating *HOTAIR* levels was affirmed in a murine model of experimental autoimmune encephalomyelitis, and in human monocytic THP-1 cells challenged with immunoactivators (recombinant human granulocyte–macrophage colony-stimulating factor and phytohaemagglutinin) (Ref. 40). In agreement with these results, sulphasalazine, another immunosuppressive drug prescribed for MS patients, was observed to appreciably repress *HOTAIR* levels through the miR-136-5p pathway. For this study, a mouse model of cuprizone-induced demyelination was employed; wherein sulphasalazine treatment stimulated oligodendrocyte differentiation and remyelination by blocking the microglial switch to pro-inflammatory M1-like phenotype. These sulphasalazine-induced protective effects were reported to be dependent on alterations in miR-136-5p sponging activities of *HOTAIR* and via modulation of the Akt2-NF-*κ*B cascade (Ref. 41). Recently, increased levels of *HOTAIR* lncRNA were reported in PBMCs of relapse-remitting MS patients in the relapse phase, making it a possible biomarker for distinguishing the remit and relapse phases (Ref. 42). Finally, it has been suggested that the rs4759314 single-nucleotide polymorphism (SNP) in *HOTAIR* may be linked with MS pathogenesis, at least in Iranian population (Ref. 43). Further studies however are warranted to confirm this in subjects from other ethnicities.

### Traumatic, hypoxic and ischaemic brain injury

Research evaluating the molecular mechanisms linking lncRNAs and the (patho)physiology of brain insults, such as stroke and traumatic brain injury (TBI), has only been recently instigated (Ref. 44). In this regard, Cheng *et al*. were the first to elucidate the pro-neuroinflammatory actions of *HOTAIR* in TBI. For this, they used an in vivo Feeney's free-fall impact model of TBI, as well as a lipopolysaccharide-challenged cellular (microglial BV2 cells) model. They showed a drastic upsurge in *HOTAIR* expression in both these pathological systems which resulted in enhanced production of inflammatory mediators, such as TNF-α, IL-1*β* and IL-6 from microglia. These effects were attributed to increased stability of myeloid differentiation primary response protein (MyD88), a protein with known pro-inflammatory effects, by repressing its ubiquitination mediated by E3 ubiquitin-protein ligase Nrdp1. Correspondingly, shRNA-mediated silencing of *HOTAIR* normalized Nrdp1 levels and prevented microglial activation (Ref. 18).

Middle cerebral artery occlusion (MCAO)-mediated hypoxia induction in rodents has been shown to result in significant increases in *HOTAIR* expression in the lesion sites, in turn culminating into elevated serum levels of nicotinamide adenine dinucleotide phosphate or NADPH oxidase 2 (NOX2). NOX2 is a primary pathogenic player in ischaemic injury and stroke was also shown to be induced in a *HOTAIR*-dependent manner in HT22 cells challenged with hypoxic insults. Moreover, *HOTAIR*–NOX2 interactions were critical in inducing apoptotic death of hypoxic cells, as evidenced by RNA interference (against *HOTAIR*) experiments (Ref. 45). Recently, Huang *et al*. have implicated the *HOTAIR*–miR-148a-3p–Kruppel-like factor 6 (KLF6) axis in neuronal death following ischaemic stroke. Their analyses in a murine MCAO model and a cellular oxygen-glucose deprivation (OGD) model provided robust affirmation of the participation of *HOTAIR* in the induction of inflammation and apoptotic death in a signal transducer and activator of transcription 3 (STAT3) pathway-dependent manner. The sponging of the miR-148a-3p–KLF6 pathway by *HOTAIR* was reaffirmed by shRNA-mediated knockdown of the latter in OGD-induced N2A cells and in mice induced with ischaemic stroke. Interestingly, knockdown of *HOTAIR* in MACO mice was associated with significant attenuation in the severity of neurological deficits and in cognitive decline, as measured by a battery of behavioural assays evaluating memory, motor, sensory and proprioceptive functions in mice (Ref. 46). In addition, sponging of miR-211 may also partly explain the pathogenic roles of *HOTAIR* in cerebral ischaemia–reperfusion injury (Ref. 47).

Similarly, human brain microvascular endothelial cells challenged with to OGD/reperfusion insult have been found to elicit robust upregulation of *HOTAIR* transcripts. Further, the association of *HOTAIR* with EZH2/PRC2 seems to be instrumental in enhancing cellular migration and in inducing membrane disintegration and pro-apoptotic pathways in the pathogenesis of endothelial dysfunction. Indeed, the roles of the *HOTAIR*–EZH2 signalling in inducing blood brain barrier (BBB) deficits are supported by marked enhancements in the plasma *HOTAIR* levels in newborn human cases of hypoxic-ischaemic encephalopathy (Ref. 48). Finally, in an interesting recent study, Ali *et al*. estimated the changes in serum *HOTAIR* levels in control subjects and cases with cerebrovascular stroke (with and without comorbidities of chronic hypertension). Surprisingly, they observed reduced *HOTAIR* expression in cerebrovascular stroke subjects, compared with the controls. Further, hypertensive group of cerebrovascular stroke cases elicited significantly lowered plasma *HOTAIR* levels when compared with their non-hypertensive counterparts. Importantly, the authors observed significant positive correlation between *HOTAIR* expression and the scores obtained on the National Institutes of Health Stroke Scale, which measures different aspects of behavioural and cognitive attributes (e.g. motor and sensory function, language and speech production, vision, attention and focus) (Ref. 49).

### Neuropsychiatric disorders

The association of rs1899663, an intronic SNP of *HOTAIR*, was evaluated in multiple psychiatric conditions, *viz.* bipolar disorder types I and II, drug (methamphetamine) addiction, major depressive disorder (MDD), schizophrenia and attention-deficit hyperactive disorder (ADHD). Although the investigators did not observe any significant connections between the rs1899663 SNP and the risk of developing schizophrenia or methamphetamine addiction, a significant association was observed for bipolar disorder type I in the allelic, co-dominant and dominant models. Further, the SNP was linked with enhanced risk of developing bipolar disorder type II, MDD and ADHD (Ref. 50), indicating that *HOTAIR* may be linked to several of the neuropsychiatric conditions. In accordance, based upon a study conducted in bipolar disorder cases and age-matched normal controls, *HOTAIR* SNPs rs1899663 G/T, rs4759314 A/G, rs12826786 C/T and rs920778 C/T have been proposed as risk factors for the development and progression of bipolar disorder. Specifically, although GT genotype of rs1899663 G/T, CT genotype of rs920778 C/T and CT genotype of rs12826786 C/T SNPs were reported to enhance the risk for bipolar disorder development, GG genotype of rs4759314 A/G SNP was shown to lessen the chances of developing the disease (Ref. 51).

Lastly, genetic studies have also implicated *HOTAIR* in the pathogeneses of autism spectrum disorders (ASDs). Thus, from the clinical data of 427 ASD subjects and 430 controls, the rs12826786 SNP in *HOTAIR* showed significant associations with the ASD-resembling phenotype in allelic (T versus C) and recessive (TT versus TC + CC) models (Ref. 52). Although these data indicate an interactive effect of *HOTAIR* SNPs on the pathogenesis of multiple psychosocial conditions across different age clusters, further research is warranted for elucidation of the functional roles of these *HOTAIR* SNPs.

### Chemically induced cognitive dysfunction

Sevoflurane, a volatile anaesthetic induces cognitive dysfunction upon prolonged exposure in humans and rodents. Employing a rat model, Wang *et al.* demonstrated that sevoflurane exposure results in appreciable loss of working and reference memory functions, as assessed by radial arm maze test; as well as a pronounced diminishment of BDNF expression. These effects were possibly mediated by RE-1 silencing transcription factor, under the control of *HOTAIR* signalling. Indeed, siRNA-mediated knockdown of *HOTAIR* was found to prevent the detrimental effects of sevoflurane on cognition and BDNF signalling (Ref. 53). Similar findings were reported for isoflurane (another volatile anaesthetic)-mediated oxidative dyshomoeostasis, neuroinflammation, cellular apoptosis and cognitive impairment in rats; wherein the investigators provided evidence for the involvement of repressed miRNA-129-5p functions mediated by *HOTAIR* as a critical pathogenic trigger (Ref. 54).

### Diabetic retinopathy and neuropathy

Severe hyperglycaemic conditions in diabetes are associated with damage to nerves (diabetic neuropathy) and retina (diabetic retinopathy). A pathogenic role for *HOTAIR* has recently been proposed for diabetic retinopathy. Thus, in a cellular model of human retinal endothelial cells exposed to high concentrations of extracellular glucose, Biswas *et al*. observed appreciable increments in *HOTAIR* levels. This was linked with detrimental alterations in redox signalling, mitochondrial bioenergetics and angiogenesis. Moreover, serum and vitreous humour of diabetic neuropathic human subjects elicited high expressions of *HOTAIR* lncRNA, as did the retinal tissues from a streptozotocin-induced rodent model of diabetic neuropathy. Lastly, RNA interference-mediated knockdown of *HOTAIR* was found to prevent many of the effects induced by hyperglycaemia (Ref. 55). SNP expression studies in human subjects have also implicated *HOTAIR* lncRNAs in the (patho)physiology of diabetic retinopathy. From the data of a case-control clinical study involving 276 individuals with diabetic retinopathy and 452 control subjects, three *HOTAIR* SNPs, rs1899663-TT, rs12427129-CT + TT and rs12427129-CT were evidenced to have abnormally elevated expression in the human cases of diabetic retinopathy, compared with the controls. In addition, significant upregulation of rs1899663-TT and rs12427129-CT + TT SNPs were observed in patients diagnosed with proliferative diabetic retinopathy (Ref. 56).

### Gliomas and other brain cancers

Gliomas are the most prevalent of the primary brain cancers and are notoriously problematic to diagnose, classify and treat (Ref. 57). Recent years have seen exponential increases in research studies evaluating the positive and negative roles of different lncRNA species, including *HOTAIR* in the (patho)physiology of gliomas (Ref. 58). Amongst the initial studies evaluating the roles of *HOTAIR* lncRNAs in human glioma subjects, Zhang *et al*. proposed that *HOTAIR* could be employed as a suitable biomarker for identification of the diverse molecular subtypes of gliomas (Ref. 59). In addition, they envisioned as a tumour grade marker, since its expression was observed to elicit robust positive correlation with tumour grade; i.e. higher *HOTAIR* levels in high-grade gliomas and conversely lower levels in low-grade gliomas. Further, based upon correlational Kaplan–Meier survival curve analysis, they suggested *HOTAIR* as a potent prognostic marker for predicting survival of glioma patients (Ref. 59). In an interesting study, *HOTAIR* gene expression, methylation status and copy number were evaluated in human glioma cases; and it was observed that high expression of *HOTAIR* was associated with higher glioma grades. An important outcome of this study was the observation of a strong correlation of *HOTAIR* expression and the levels of homoeobox protein HOXA9, particularly in higher-grade gliomas; compelling the authors to undertake chromatin immunoprecipitation-quantitative polymerase chain reaction analyses. This led the authors to identify a direct role of HOXA9 in binding to the promoter and regulating the expression of *HOTAIR* during glioma pathogenesis (Ref. 60). In concurrence with these results, upon microarray-based assessment of the altered expression levels of lncRNA species in human glioblastoma patients versus controls, *HOTAIR* was established as one of the major lncRNAs with significant upregulations in glioblastoma (Ref. 61). Similarly, analyses of the RNA-seq data from Chinese glioma subjects identified *HOTAIR* as a key overexpressed lncRNA (Ref. 62). These results are in concurrence with those obtained by Lv *et al*. who employed The Cancer Genome Atlas (TCGA) database for identification of *HOTAIR*, *LOC00132111* and *DLEU1* as the lncRNAs species with the strongest associations with the overall survival rate of glioma cases (Ref. 63).

In silico (and combinatorial) studies have been instrumental in our understanding of the multimodal tumour-promoting roles played by *HOTAIR* in gliomas. For example, Huang *et al*. employed the Chinese Glioma Genome Atlas and proposed a cell cycle-related mRNA network made up of 18 genes positively regulated by *HOTAIR* as the latter's core feature promoting proliferation of glioma cells (Ref. 64). Similarly, using RNA sequencing data from TCGA, Lei *et al.* recognized *HOTAIR* as one of the most conspicuous lncRNAs as a biomarker for glioblastoma prognosis and predictor of patient survival (Ref. 65). Combining data for lncRNA and gene expression profiles, with transcription factor–gene target relationships in computational frameworks, Li *et al*. identified multitudes of the lncRNA–transcription factor–gene target triplet axes with significant associations with glioblastoma (patho)physiology. Some of these included the *HOTAIR*–MAX-interacting protein 1–CD58/protein kinase C epsilon type (HOTAIRMXI1–CD58/PRKCE) and the *HOTAIR*–activating transcription factor 5–neural cell adhesion molecule 1 (*HOTAIR*–ATF5–NCAM1) axes. The data indicated that *HOTAIR* and the abovementioned targets may serve as potent targets for diagnosis, molecular classification, therapy, prognosis and survival-predictors for gliomas in humans (Ref. 66). Data from patients diagnosed with glioblastoma or low-grade gliomas (LGGs) obtained from the TCGA and Gene Expression Omnibus databases have been recently used to create a ceRNA network for distinction between glioblastoma and LGGs, and *HOTAIR* was amongst 13 lncRNAs identified as hub components with the highest degrees of connections in this ceRNA network (Ref. 67). Other analytical studies focusing on the altered expression of lncRNAs in glioblastoma pathogeneses using data from TCGA databases have identified *HOTAIR* as a major lncRNA with exceedingly high levels of expression in the diseased subjects (Ref. 68). Gene ontology analyses indicated that *HOTAIR* influences key physiological cascades such as cell proliferation and cell cycle progression, and DNA and RNA metabolism (Ref. 69). Small nucleolar RNA, C/D box small nucleolar RNA 76 (SNORD76) was proposed as novel target of *HOTAIR* signalling in glioblastoma pathology by Chen and coworkers. They found that *HOTAIR* reduces the expression of SNORD76, resulting in retinoblastoma gene-induced cell cycle arrest in glioma cells. *HOTAIR*-induced reduction in SNORD76 expression in tumour tissues supported the former's tumour-stimulatory role in glioblastoma (Ref. 70). The tumour-promoting functions of *HOTAIR* in glioma (patho)physiology may also depend on its capability to repress the functions of programmed cell death protein 4 (PDCD4) (Ref. 71). More recently, it has been demonstrated that pro-metastatic actions of *HOTAIR* rely on pathogenic alterations in Nemo-like kinase (NLK) signalling, with consequent overactivation of the *β*-catenin pathway (Ref. 72).

*HOTAIR*'s capability to act as a molecular sponge and repress a plethora of target miRNAs (Table 1) adds another facet to its pro-tumourigenic roles in glioma pathology. Indeed, data obtained from human glioma tissues as well as U251 and U87 glioma cell lines show aberrant overexpression of *HOTAIR* and the consequent trans-silencing of miR-141. This *HOTAIR*–miR-141–spindle and kinetochore-associated protein 2 (SKA2) signalling axis was proposed to be instrumental in inhibition of tumour xenograft growth in mice based upon experiments performed to both knockdown *HOTAIR* and overexpress miR-141 (Ref. 73). miR-326 is another target of *HOTAIR* which is implicated in gliomas. miR-326 sponging and the consequent tumour-promoting actions of *HOTAIR* may stem from hyperactivation of fibroblast growth factor 1 (FGF-1) signalling, causing hyperactivation of phosphoinositide 3-kinase/Akt and mitogen-activated protein kinases 1/2 signalling cascades (Ref. 74). Using an A172 glioma cell line, miR-148b-3p was identified to be another direct mediator of pro-tumourigenic effects of *HOTAIR* lncRNA in gliomas. Thus, *HOTAIR* expression was observed to be inversely related to that of miR-148b-3p; and its si*HOTAIR*-mediated silencing led to attenuation of survival, malignant, proliferative and invasive abilities of the A172 cells (Ref. 75). Interestingly, the *HOTAIR*–miR-148b-3p–upstream stimulatory factor 1 (USF-1) pathway has been proposed as a focal point regulating the permeability of blood tumour barrier (BTB). *HOTAIR* expression was found to be heavily upregulated in a cellular model of BTB fabricated as a co-culture of human cerebral microvascular endothelial cell line hCMEC/D3 and human glioblastoma U87 cells. This was paralleled by downregulation of miR-148b-3p expression, and upregulation in USF-1 levels, resulting in altered expressions of tight junction forming proteins (zonula occludens-1, occludin and claudin-5) in glioma microvascular endothelial cells (Ref. 76). miR-301a-3p may represent another sponging target of *HOTAIR* in gliomas, and inhibition of miR-301a-3p mediated by *HOTAIR* has been reported to cause induction of the expression of transcription factor, Fos-like 1 (FOSL1) (Ref. 77). The miR-126-5p–glutaminase (GLS) axis is yet another target of *HOTAIR* in the pathogeneses of gliomas. The ability of *HOTAIR* to act as a ceRNA for miR-126-5p and promote GLS expression was observed in human tissue samples from cases of glioblastomas, astrocytomas and oligodendrogliomas (Ref. 78). In addition to confirming the pro-cancer actions of *HOTAIR*, the study, for the first time, provided evidence for the engrossment of *HOTAIR* in the modulation of glutamine metabolism. Further, *HOTAIR* has been reported to modulate the miR-15b/p53 regulatory loop which regulates proliferation, growth and metastasis of glioma cells. Thus, although overexpression of miR-15b and p53 promoted apoptosis and inhibited proliferation and invasion of human glioma U87 cells, *HOTAIR* upregulation culminated into enhanced proliferativity and invasivity of these cells (Ref. 79). The miR-218–phosphodiesterase 7A (PDE7A) axis is also thought to be a target of *HOTAIR* with regards to glioma (patho)physiology (Ref. 80). Experiments with both glioma tissues and cell lines confirmed high expression levels of *HOTAIR* lncRNA, concomitantly with significant repressions in miR-218 levels. Further, experimental data suggest that altering *HOTAIR*–miR-218–PDE7A cascade by shRNA-mediated *HOTAIR* inhibition or miR-218 overexpression results in apoptotic induction and suppression of proliferation and invasion in U251 glioma cells.

**Table 1.** Involvement of genetic variants of *HOTAIR* SNPs in neuropathological conditions

Sl. no.	SNPs of *HOTAIR*	Pathological condition	Ref.
1	rs1899663	Bipolar disorders	50, 51
		MDD	50
		ADHD	50
		Diabetic retinopathy	56
2	rs4759314	MS	43
		Bipolar disorders	51
3	rs12826786	Bipolar disorders	51
		ASDs	52
		Grade III anaplastic oligodendroglioma	81
4	rs920778	Bipolar disorders	51
		Grade III anaplastic oligodendroglioma	81
5	rs12427129	Diabetic retinopathy	56

SNPs of *HOTAIR* have been linked to multiple neuropathologies, including MS, neuropsychiatric conditions and brain cancers (Table 2). One of the initial studies which assessed the associations of *HOTAIR* polymorphisms in glioma pathogenesis was conducted by Xavier-Magalhães *et al*. in Portuguese subjects diagnosed with multiple types of gliomas. Interestingly, although the authors reported the absence of any statistically significant changes in the distribution of the rs920778 or the rs12826786 *HOTAIR* SNPs between the cases and controls, both rs12826786 CT and rs920778 CT genotypes were found to be robustly associated with the survival of grade III anaplastic oligodendroglioma subjects, indicating the probable benefits of using these SNPs as prognosis markers for this particular glioma type (Ref. 81). Needless to say, more research data are required to establish and confirm the linkages of novel SNPs in *HOTAIR* with glioma (patho)physiology.

**Table 2.** Molecular sponging targets of *HOTAIR* in neuronal disorders

Sl. no.	Signalling axis	Pathological condition	Ref.
1	*HOTAIR*–miR-15/107–CDK5 (cyclin-dependent kinase 5)	AD	26
2	*HOTAIR*–miR-126-5p–RAB3IP (RAB3A interacting protein)	PD	34
3	*HOTAIR*–miR-221-3p–NPTX2 (neuronal pentraxin II)	PD	35
4	*HOTAIR*–miR-326–ELAVL1 (ELAV-like RNA binding protein 1)	PD	36
5	*HOTAIR*–miR-874-5p–ATG10 (autophagy-related protein 10)	PD	37
6	*HOTAIR*–miR-136-5p–Akt2–NF-*κ*B	MS	41
7	*HOTAIR*–miR-148a-3p–KLF6 (Kruppel-like factor 6)	Ischaemic stroke	46
8	*HOTAIR*–miR-141–SKA2 (spindle and kinetochore-associated protein 2)	Glioma	73
9	*HOTAIR*–miR-326–FGF-1 (fibroblast growth factor 1)	Glioma	74
10	*HOTAIR*–miR-148b-3p–USF-1 (upstream stimulatory factor 1)	Glioma	76
11	*HOTAIR*–miR-301a-3p–FOSL1 (Fos-like 1)	Glioma	77
12	*HOTAIR*–miR-126-5p–GLS (glutaminase)	Glioma	78
13	*HOTAIR*–miR-218–PDE7A (phosphodiesterase 7A)	Glioma	80
15	*HOTAIR*–miR125–HK2 (hexokinase 2)	Glioblastoma	85
16	*HOTAIR*–miR-526b-3p–EVA 1 (epithelial V-like antigen 1)	Glioblastoma	83
17	*HOTAIR*–miR-483-3p–CDK4 (cyclin-dependent kinase 4)	Medulloblastoma	87
18	*HOTAIR*–miR-1/206–YY1 (Yin Yang 1)	Medulloblastoma	88
19	*HOTAIR*–miR-125a–mTOR (mammalian target of rapamycin)	Glioma	96

Studies have also implicated *HOTAIR* signalling as a major molecular agent conferring chemoresistance to cancer cells against multiple chemotherapeutics. With regards to CNS cancers, cisplatin has been shown to retard EMT in glioblastoma and neuroblastoma cells, possibly by its ability to alter *HOTAIR* signalling (Ref. 82). Interestingly, exposure of *HOTAIR*-containing extracellular vesicles obtained from the serum samples of glioblastoma subjects has been observed to tremendously expedite glioma proliferation and temozolomide-resistance in vitro (U251 and LN229 cells) and in vivo (orthotopic transplantation of U251 cells in mice brain). This temozolomide-resistance conferred by extracellular vesicular *HOTAIR* was possibly because of the diminution of inhibitory actions of miR-526b-3p on epithelial V-like antigen 1 (EVA 1), a regulator of programmed cell death cascades (Ref. 83). In U251 glioma cells, *HOTAIR* regulatory element was found to determine sensitivity to temozolomide, possibly via long-range expressional alteration of target genes, *CALCOCO1* (calcium binding and coiled-coil domain 1) and *ZC3H10* (zinc finger CCCH-type containing 10) (Ref. 84). Moreover, temozolomide-resistance in gliomas may manifest because of aberrant overactivation of hexokinase 2 (HK2) and the glycolytic pathway in a *HOTAIR*–miR125-reliant manner. Indeed, *HOTAIR* repression was found to result in strong downregulation of HK-2 mRNA and protein, and in enhanced susceptibility of glioma cells to temozolomide (Ref. 85).

In addition to gliomas, *HOTAIR* has been implicated in the pathogeneses of other brain tumours. Significant upregulation of *HOTAIR* has been observed in tissues from atypical teratoid rhabdoid tumours, medulloblastomas and juvenile pilocytic astrocytomas (Ref. 86). A more recently published study attempted to discern the molecular mechanisms of *HOTAIR*-mediated tumourigenic effects in medulloblastoma cells. shRNA-mediated silencing of *HOTAIR* was found to result in a significant induction of cellular apoptosis and attenuation of proliferative abilities of medulloblastoma cells, possibly via altered regulation of the miR-483-3p–CDK4 axis (Ref. 87). According to Zhang *et al*., the *HOTAIR*–miR-1/miR-206–Yin Yang 1 (YY1) axis may also be involved in the pathogeneses of medulloblastomas (Ref. 88). A recent study has evaluated the role of *HOTAIR* lncRNA in the pathology of a variety of ependymomas, and revealed its significantly elevated expression in myxopapillary ependymoma (MPE), particularly spinal MPE, when compared with other spinal and intracranial ependymomas. Since, its expression was unaltered in non-ependymoma tumours of the spinal cord, the authors proposed that *HOTAIR* may serve as an effective and specific diagnostic marker of spinal MPE (Ref. 89).

## *HOTAIR* as a diagnostic marker and bio-therapeutic target

### Diagnostic and prognostic potential of *HOTAIR*

Several studies have indicated the potential usage of *HOTAIR* as an indicator of poor prognosis in high grade gliomas. Indeed, circulating lncRNAs such as *HOTAIR* may represent a novel and relevant category of prognoses of gliomas (Ref. 68). Shen *et al*. assessed the levels of known tumourigenic lncRNAs (including *HOTAIR*) in serum samples of primary glioblastoma cases and evaluated their associations with outcomes. For *HOTAIR*, there was a sturdy and positive correlation with progression and relapse of the condition, and with increased likelihood of mortality, with the adjusted hazard ratios estimated at 1.82 and 2.04; respectively (Ref. 90). *HOTAIR* expression in exosomes derived from serum may also be a potent peripherally acquired biomarker for glioma diagnoses and prognoses. In the study by Tan *et al*. for example, the exosomal expression of *HOTAIR* was assessed in serum samples isolated from 43 subjects of glioblastoma multiforme and 40 control subjects. Pearson's correlational analyses indicate a significant correlation between *HOTAIR* levels in serum-derived exosomes and in the corresponding tumour tissues. Importantly, for the serum exosomal *HOTAIR* expression levels, the area under the receiver operating characteristic (ROC) curve significantly differentiated the subjects from the controls with an estimated value of 0.913, and with high sensitivity (86.1%) and specificity (87.5%), indicating their suitability as a peripherally sourced minimally invasive prognostic and diagnostic biomarker (Ref. 91). Along similar lines, using a mouse xenograft model comprising transplanted malignant glioma U87 cells, Ren *et al*. have advocated for the potential safety, specificity and effectiveness of liposome-encapsulated radiolabelled antisense oligonucleotide probes against *HOTAIR* for the in vivo real-time imaging of *HOTAIR*-positive gliomas (Ref. 92). Although the relevance of peripheral levels of *HOTAIR* for diagnostic and prognostic purposes in oncology is well-established, research studies are warranted to extend this possibility to other neuropathologies.

### Therapeutic strategies based upon *HOTAIR* signalling

Considering its extensive multifaceted pro-tumourigenic roles, numerous groups have suggested that *HOTAIR* may be a potential target for novel anti-glioma therapeutic strategies. Indeed, in recent years, several therapeutic agents and approaches have been proposed which are based upon repression of *HOTAIR* functions. For instance, a captivating RNA-based strategy for suppression of pro-tumourigenic actions of *HOTAIR* has been proposed. The authors of this study constructed a dominant negative deletion mutant of *HOTAIR* which was incapable of binding and recruiting EZH2 protein. The mutant *HOTAIR* was able to effectively abolish the tumourigenic actions (such as promotion of cellular invasivity and EMT) of endogenous *HOTAIR*, both in vitro and in vivo (Ref. 93). Interestingly, *HOTAIR* has also been proposed as a key target of oncolytic virus-mediated glioma therapy. Thus, Vazifehmand *et al.* provided evidence for the alterations in the levels of *HOTAIR* (amongst other lncRNA species) upon treatment of glioblastoma U251 cells with HSV-G47Δ oncolytic viral particles (Ref. 94). In a very interesting recently published study, clustered regularly interspaced short palindromic repeat (CRISPR)-based interference was employed to identify the lncRNAs, including *HOTAIR* which undergo massive expressional changes during the progression of gliomas in vivo, for example in a mouse xenograft model of glioblastoma U87 cells transplanted in the cerebral cortices (Ref. 95). Further, Schisandrin B, a bioactive phytochemical from the Chinese medicinal plant *Schisandra chinensis*, was demonstrated to harbour significant anti-glioma (apoptotic induction, and reduced cellular viability, migration and invasiveness) potential, at least in part, by repressing the sponging actions of *HOTAIR* against miR-125a–mTOR (mammalian target of rapamycin) signalling (Ref. 96).

Although these studies indicate the possible utilities of *HOTAIR*-based therapeutic strategies in gliomas and other cancers, further studies are required to ascertain and extend the utility of such strategies in the clinical settings. In this regard, novel smart nanoplatforms may serve as trend setters (Ref. 57). Indeed, magnetic nanoparticles loaded with siRNA against *HOTAIR* have been found to efficiently repress its expression, resulting in robust retardation of cell proliferation and invasiveness. This si-*HOTAIR* containing magnetic nanotherapeutic agent was also shown to cause significant anti-tumourigenic effects in an in vivo model of xenograft of glioma cells in nude mice, presumably because abolishment of *HOTAIR*–EZH2/LSD1 signalling resulted in stimulated expression of PDCD4 as well as decreases in the expression of cell cycle and growth regulators; cyclin D1, cyclin-dependent kinase 4 (CDK4), BDNF and Ki67 (Ref. 97). It will be interesting to study the therapeutic implications of similar smart nanoformulations against other brain pathologies.

**Figure 4.**
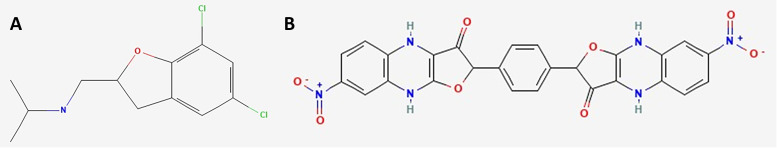
Small-molecular inhibitors of *HOTAIR* signalling. 2D structures of (A) (*N*-[(5,7-dichloro-2,3-dihydro-1-benzofuran-2-yl)methyl]propan-2-amine), also known as AC1Q3QWB (AQB; CID 36806), and (B) 7-nitro-2-[4-(7-nitro-3-oxo-4,9-dihydrofuro[3,2-b]quinoxalin-2-yl)phenyl]-4,9-dihydrofuro[3,2-b]quinoxalin-3-one, also known as AC1NOD4Q (ADQ; CID 5086250). The structures are obtained from PubChem (https://pubchem.ncbi.nlm.nih.gov/substance/117467309#section=2D-Structure and https://pubchem.ncbi.nlm.nih.gov/substance/113734165#section=2D-Structure).

Using molecular docking simulations, Li *et al*. identified AC1Q3QWB (*aka* AQB; Fig. 4A) as a probable small-molecule inhibitor of *HOTAIR*–EZH2–PRC2 signalling. Experimental analyses confirmed AQB's robust inhibitory actions on *HOTAIR*, and the consequent upregulation of tumour suppressor gene, antigen-presenting cell (APC) regulator of WNT signalling pathway 2 and repression of Wnt/*β*-catenin cascade in primary patient-derived glioblastoma cells, N5 and N33. Further, when complemented with 3-deazaneplanocin A (DZNep), an EZH2 inhibitor, AQB resulted in much better therapeutic actions (than DZNep alone) in vitro and in vivo (xenograft glioblastoma rodent models) (Ref. 98). In a subsequent study, AQB was found to elicit increments in the levels of tumour suppressor genes, such as the CWF19-like cell cycle control factor 1 which in turn resulted in degradation of CDK4/6, and cell cycle arrest at G1 stage. In fact, synergistic actions of AQB and CDK4/6 inhibitor palbociclib were found to induce robust anti-metastatic effects both in vitro (U87 and N33 cells) and in vivo (glioblastoma xenograft model in mice), possibly via inhibition of the hyperactive Wnt/*β*-catenin signalling (Ref. 99). In their subsequent study, the research group proposed programmed death ligand 1-reliant induction of NF-*κ*B signalling as a key *HOTAIR*-based cellular target against immune tolerance in glioma therapy. They observed that AQB treatment caused significant attenuation of upregulated levels of inflammatory markers (*viz.* NF-*κ*B, IL-8, IL-1*β* and TNF-*α*) in U87 and TBD glioma cells. Further analyses involving AQB-dependent repression of *HOTAIR* signalling indicated the possible involvement of ubiquitin regulatory X domain protein 1 in these effects. Importantly, AQB-induced inhibition of *HOTAIR* resulted in enhanced immune sensitivity of glioma cells, therefore causing significant reduction in the tumour load and enhancement of survival time of nude mice confronted with orthotopic glioma cell transplant (Ref. 100). A combinatorial therapeutic strategy involving AQB and LSD1 inhibitor GSK-LSD1 was proposed as an efficient anti-glioma therapeutic strategy in vitro and in patient-derived xenograft models. Since, *HOTAIR*–EZH2 interactions require a functional 5′-domain of *HOTAIR* and binding of LSD1 to *HOTAIR* relies on the latter's 3′-domain, complemented application of AQB and GSK-LSD1 were found to result in robust blocking of both the functional domains of *HOTAIR*, culminating into significant inhibition of cell cycle progression genes, concomitantly with activation of pro-apoptotic genes (Ref. 101). Recently, Ren *et al*. have identified another specific inhibitor of the *HOTAIR*–EZH2 interaction in a high throughput molecular docking based in silico analyses. The small-molecule inhibitor, AC1NOD4Q (*aka.* ADQ; Fig. 4B) was reported to halt H3K27-methylation of NLK, a downstream target of *HOTAIR* which consequently resulted in repression of cellular metastases in a Wnt/*β*-catenin pathway-dependent manner. RNA immunoprecipitation and electrophoretic mobility shift assay experiments further indicated that ADQ binds to the 5′-domain of *HOTAIR* which is directly involved in binding and recruitment of EZH2 (Ref. 102).

## Conclusions

Although it is clear that *HOTAIR* is a pro-oncogenic lncRNA which detrimentally affects the pathogeneses of gliomas at multiple levels and via a plethora of molecular targets, its pathogenic impact during the initiation and progression of other neuronal dysfunctions has largely remained undiscerned. To this end, this review attempted a comprehensive summarization of all research data implicating *HOTAIR* in different neuropathologies. We hope that there will be more research studies focused on understanding the molecular and cellular mechanisms underlying the (patho)physiological actions of *HOTAIR* in the brain. We also direct the attention of the readers to relevance of *HOTAIR* as a particularly interesting marker for disease diagnosis/prognosis, as well as a bio-target for the design of therapeutic strategies against CNS pathologies, including brain cancers. Of course, there are significant challenges which have to be addressed before *HOTAIR* can be effectively used as a diagnostic and prognostic biomarker or a therapeutic biotarget for neuronal disorders under the clinical settings. First, the molecular mechanisms underlying *HOTAIR*-mediated detrimental effects must be characterized comprehensively in appropriate and specific pre-clinical disease models. Second, analyses of *HOTAIR* polymorphisms must be extensively undertaken in order to extricate out the genetic linkages with reference to the different neuronal disorders. Third, design and evaluation of inhibitory agents/regimens have to be performed in order to ameliorate the tremendously dysfunctional *HOTAIR* signalling in CNS and other disorders. Only when these hurdles are passed, we will be able to fully exploit *HOTAIR* as a multifaceted biomarker and biotarget.

## Funding statement

The authors extend their appreciation to the Deputyship for Research & Innovation, Ministry of Education in Saudi Arabia for funding this research work through the Project Number ISP23-101.

## Abbreviations

AD, Alzheimer's disease; ADHD, attention-deficit hyperactive disorder; ADQ, AC1NOD4Q; APP, amyloid precursor protein; AQB, AC1Q3QWB; ASD, autism spectrum disorder; ATG10, autophagy-related protein 10; BDNF, brain-derived neurotrophic factor; BTB, blood tumour barrier; CDK, cyclin-dependent kinase; CNS, central nervous system; CoREST1, REST corepressor 1; DZNep, 3-deazaneplanocin A; ELAVL1, ELAV-like RNA binding protein 1; EMT, epithelial-to-mesenchymal transition; EVA 1, epithelial V-like antigen 1; EZH2, enhancer of Zeste homologue 2; FGF-1, fibroblast growth factor 1; FOSL1, Fos-like 1; GLS, glutaminase; HK2, hexokinase 2; *HOTAIR*, HOX transcript antisense intergenic RNA; IL, interleukin; KLF6, Kruppel-like factor 6; lncRNA, long non-coding RNA; LRRK2, leucine-rich repeat kinase 2; LSD1, lysine-specific demethylase 1; miRNA or miR, micro-RNA; MCAO, middle cerebral artery occlusion; MDD, major depressive disorder; Mex3b, Mex-3 RNA-binding family member B; MPE, myxopapillary ependymoma; MPP^+^, *N*-methyl-4-phenylpyridinium; MS, multiple sclerosis; mTOR, mammalian target of rapamycin; NF-*κ*B, nuclear factor kappa B; NLK, Nemo-like kinase; NOX2, NADPH oxidase 2; NPTX2, neuronal pentraxin II; OR, odds ratio; PD, Parkinson's disease; PDCD4, programmed cell death protein 4; PDE7A, phosphodiesterase 7A; PBMC, peripheral blood mononuclear cell; PRC2, polycomb-repressive complex 2; PS-1, presenilin-1; RAB3IP, RAB3A interacting protein; REST, repressor element 1-silencing transcription factor; SKA2, spindle and kinetochore-associated protein 2; SNORD76, small nucleolar RNA, C/D box small nucleolar RNA 76; SNP, single-nucleotide polymorphism; TBI, traumatic brain injury; TCGA, The Cancer Genome Atlas; TNF-*α*, tumour necrosis factor-alpha; USF-1, upstream stimulatory factor 1; YY1, Yin Yang 1
